# Rationale and Design of the Cooperative Program for ImpLementation of Optimal Therapy in Heart Failure

**DOI:** 10.1002/clc.70222

**Published:** 2026-02-06

**Authors:** Alexander J. Blood, Ozan Unlu, John W. Ostrominski, Shahzad Hassan, Hunter Nichols, Samantha Subramaniam, Daniel Gabovitch, Jacqueline Chasse, Marian McPartlin, Christian Figueroa, Emma Collins, Megan Twining, Matthew Varugheese, Kavishwar Wagholikar, Christopher P. Cannon, Akshay S. Desai, Benjamin M. Scirica

**Affiliations:** ^1^ Accelerator for Clinical Transformation, Brigham and Women's Hospital Boston Massachusetts USA; ^2^ Division of Cardiovascular Medicine Brigham and Women's Hospital Boston Massachusetts USA; ^3^ Harvard Medical School Boston Massachusetts USA; ^4^ Department of Biomedical Informatics Harvard Medical School Boston Massachusetts USA; ^5^ Division of Endocrinology Diabetes and Hypertension, Brigham and Women's Hospital Boston Massachusetts USA; ^6^ Division of Pharmacy Brigham and Women's Hospital Boston Massachusetts USA; ^7^ Research Information Science and Computing, Mass General Brigham Somerville Massachusetts USA

**Keywords:** digital health, guideline‐directed medical therapy, heart failure, remote health

## Abstract

**Background:**

Despite overwhelming evidence of clinical benefit for patients with heart failure (HF), the uptake of guideline‐directed medical therapies (GDMT) has been slow. Collaborative approaches are critically needed to improve alignment between evidence and clinical practice. Many strategies proposed to improve GDMT implementation have been either ineffective or too resource‐intensive to implement at scale across different practice contexts. Furthermore, most existing approaches focus primarily on patients with HF and reduced EF, despite growing evidence for effective pharmacologic therapy in those with HF and mildly reduced or preserved ejection fraction (HFpEF).

**Hypothesis:**

Based on this experience, we designed the Cooperative Program for ImpLementation of Optimal Therapy in Heart Failure (COPILOT‐HF) study (NCT05734690).

**Methods:**

This is a pragmatic, randomized, open‐label intervention trial to compare a comprehensive, remote, navigator‐led, algorithm‐driven strategy for optimization of GDMT prescribing in patients with HF across the full spectrum of ejection fraction with a control intervention focused on patient and provider education regarding the importance of GDMT optimization.

**Results:**

The primary efficacy endpoint of the study is the proportion of patients receiving optimal HF treatment at 3 months. Additional outcomes of interest include the proportion of patients with optimal HF therapy at 6 months and 12 months as well as health resource utilization, including hospitalizations and deaths.

**Conclusions:**

COPILOT‐HF will evaluate the effectiveness of an early implementation of a remote pharmacist‐led medication titration strategy across the HF spectrum.

## Graphical abstract

The COPILOT‐HF trial is a pragmatic, randomized study designed to determine if a remote, pharmacist‐led navigator‐driven intervention with patient and provider education is more effective than patient and provider education alone at improving the implementation of guideline‐directed medical therapy for patients across the full spectrum of heart failure.

## 1 Introduction

The last two decades have seen dramatic advances in the development of effective pharmacologic therapies for patients with heart failure (HF) across the full spectrum of left ventricular ejection fraction (LVEF) [1]. Current guidelines from the ACC/AHA/HFSA and from the ESC recommend treatment of all patients with symptomatic HF and reduced EF (LVEF < 50%) with a four drug regimen including an evidence‐based beta blocker (BB), an angiotensin receptor neprilysin inhibitor (ARNI) (in preference to an ACE inhibitor or ARB), mineralocorticoid receptor antagonist (MRA), and sodium‐glucose co‐transporter 2 inhibitor (SGLT2i) [2]. Although evidence‐based recommendations for patients with HF and higher EF have historically been lacking, newer data supports extension of the same four drug therapy to patients with HF and mildly reduced ejection fraction (LVEF 41%–49%), and use of SGLT2 inhibitors for patients with symptomatic HF regardless of EF (including patients with HF and mildly reduced or preserved ejection fraction [HFpEF], LVEF ≥ 50%) [3, 4]. [2, 5]. Growing endorsement by guidelines of pharmacologic treatment for HF across the full spectrum of EF heightens the urgency for clinicians to deploy these treatments in clinical practice.

Despite the evolution of guidelines based on compelling evidence from randomized clinical trials, utilization of recommended therapies remains suboptimal in clinical practice [6, 7], with implications for preventable death, disability, and healthcare‐related costs. Registry data from the US and Europe suggest that one in three eligible patients with HFrEF is missing treatment with one or more key recommended therapies, and for those who are treated, underdosing is common. Although utilization of newer therapies such as ARNI and SGLT2i might be anticipated, rates of MRA utilization as low as 30% suggest major lingering implementation gaps [6, 7]. Although registry data regarding treatment of H patients with mildly reduced or preserved EF is limited, recent clinical trials enrolling this population suggest that less than one in five eligible patients is treated with an SGLT2i [8].

Several strategies have been developed to close the implementation gap between evidence and practice for HF patients. Trials of timely alerts delivered via electronic health records (EHRs) [9], virtual inpatient care‐teams [10] to nudge providers to prescribe guideline‐directed medical therapies (GDMT), and interventions to educate patients about GDMT to impact prescriber behavior [11] have demonstrated only modest efficacy and virtually all have excluded patients with HF and LVEF > 40%, who comprise a growing proportion of the population [12]. The Safety, Tolerability and Efficacy of Rapid Optimization, Helped by NT‐proBNP Testing, of Heart Failure Therapies (STRONG‐HF) trial showed that early and rapid up‐titration of GDMT significantly reduced 180‐day all‐cause mortality and HF readmissions [13]. However, owing to its resource‐intensive protocol, the scalability of the STRONG‐HF approach within the current practice environment in the United States may be challenging. Therefore, there is a substantial need for similarly effective but scalable programs to improve guideline‐directed management of HF.

We have previously implemented a successful quality improvement program utilizing a remote, navigator‐led, pharmacist‐driven strategy to facilitate medication optimization in patients with HFrEF [14]. Following that effort, newer therapies (e.g., SGLT2i), expanded regulatory labeling (e.g., ARNI in HFpEF with low‐normal EF), and revised guideline recommendations have collectively stressed the need for renewed approaches to optimize GDMT across the HF spectrum [1, 15]. Therefore, we designed the Cooperative Program for ImpLementation of Optimal Therapy in Heart Failure (COPILOT‐HF) trial to determine whether a similar remote management approach in patients with HF across the spectrum of EF will achieve a higher rate of GDMT implementation than a strategy of patient and provider education followed by remote HF clinic management. In addition, we aimed to determine, in eligible patients with left ventricular EF < 50%, whether the sequencing of GDMT initiation (traditional vs. SGLT2i‐first) leads to improved GDMT intensification. Herein, we summarize the key design elements of this trial.

## 2 Program Design

**Figure 1:**
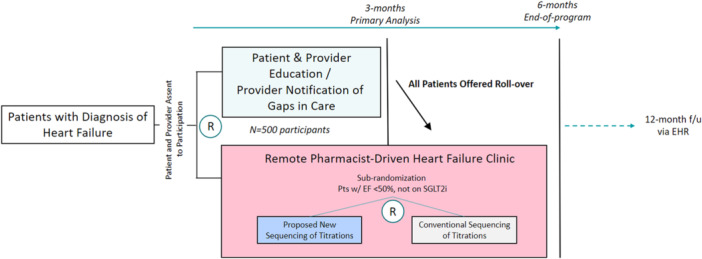
Clinical trial design.

COPILOT‐HF is a pragmatic, randomized, open‐label intervention trial to investigate the comparative effectiveness of two remote care strategies on optimizing the prescription of GDMT in patients with HF (Figure 1). The first strategy is an “education‐first” approach in which the intervention is provider and patient education with the intention of improving patients' understanding of their diagnosis and its management to increase acceptance and compliance of medical therapy. At 3 months postrandomization, this is followed by a remote clinic that implements a standardized, stepped‐approach to GDMT optimization. In the second approach, provider and patient education is started simultaneously with remote GDMT management clinic. The trial is registered at: https://clinicaltrials.gov/study/NCT05734690.

### 2.1 Patient Selection and Enrollment

Recruitment and screening will be performed within the Mass General Brigham (MGB) health system which is a nonprofit, academic healthcare system serving Massachusetts and New Hampshire and encompassing a comprehensive network that includes 2 academic medical centers (Brigham and Women's Hospital [BWH] and Massachusetts General Hospital), 12 community and specialty hospitals, and extensive network of affiliated physicians, numerous community health centers, and a dedicated home care organization. Patients aged 18–90 years with an established diagnosis of HF regardless of LVEF will be included in the study. All patients will be required to (1) have an established longitudinal relationship with a primary care provider (PCP) or a cardiologist within MGB with ≥ 1 clinic visit within 2 years before enrollment, (2) have undergone a transthoracic echocardiogram (TTE) within the 2 years before enrollment, and (3) have had symptomatic HF within the 2 years before enrollment. Criteria for determining if a patient has symptomatic HF include prior HF hospitalization, regular use of loop diuretics, or documentation of symptoms attributed to HF by the PCP or cardiologist, including but not limited to dyspnea, lower extremity edema, orthopnea, or paroxysmal nocturnal dyspnea. Detailed study inclusion and exclusion criteria can be found in Table 1.

**Table 1:** Inclusion and exclusion criteria.

Inclusion criteria	Exclusion criteria
Adults aged 18–90 years Diagnosis of ACC/AHA Stage C heart failure Current HF symptoms or symptoms within the past 24 months Most recent LVEF assessed within the past 24 months Seen a Mass General Brigham Provider within the past 24 months Within the last 24 months English or Spanish speaking	LVEF < 50% currently prescribed or intolerant to an evidence‐based beta‐blocker, ARNI, MRA, and SGLT2i at least 50% goal dose LVEF ≥ 50% currently prescribed or intolerant to SGLT2i Systolic blood pressure (SBP) < 90 mmHg at last measure Current severe aortic stenosis or severe aortic insufficiency Group 1 pulmonary arterial hypertension on disease‐specific therapies (e.g., Ambrisentan, Bosentan, Epoprostenol, Treprostinil, and Iloprost) Congenital Heart Disease Established hypertrophic cardiomyopathy with or without LVOT obstruction Type 1 diabetes Established diagnosis of cardiac amyloidosis eGFR < 30 mL/min/1.73 m^2^ Active chemotherapy Receiving end‐of‐life care or hospice History of transplant, currently listed above status 4 or being evaluated for transplant Outpatient intravenous inotrope use Current use of a ventricular assist device Currently pregnant or breastfeeding Physician's discretion is inappropriate for remote management program

Potential participants for the study are identified from several sources, including the MGB enterprise data warehouse, inpatient hospitalization dashboards, direct provider referrals, and patients previously managed by the MGB Remote Health team [14]. To identify potential MGB participants likely to meet eligibility criteria, we constructed a search query using billing codes, a clinician‐curated EHR problem list, and TTE reports for LVEF groups [16]. In addition, we implemented a best practice alert (BPA) to nudge patients' PCPs, cardiologists, and inpatient teams to augment referrals for potential enrollment. We will use summary reports of BPAs triggered for hospitalized patients and contact them postdischarge if they are eligible.

A study staff member verifies patients' eligibility. Through email or in‐basket messaging via the EHR, the program notifies potential participants' providers (cardiologists and PCPs) of the program details, their patient's eligibility, and the program's intent to invite their patient to participate. If a provider communicates that a patient is not suitable for the program, the program does not approach the patient. Eligible patients whose providers do not object are invited to participate through a secure patient gateway message. Potential participants who respond to neither the personalized letter nor the email research invitations are contacted by phone. Patients interested in participating are sent the electronic informed consent form (eConsent) via REDCap. Each patient's preferred method of communication will also be solicited and recorded by the study team. This protocol was approved by the MGB Human Research Committee.

Enrollment for the COPILOT‐HF trial has been completed. We anticipate completing follow‐up for all participants and reporting results by January 2026.

### 2.2 Randomization

Once a patient agrees to participate in the program, they are randomized in a 1:1 manner at the patient level into either (1) the “education‐first” arm (3‐month period of patient and provider education, followed by enrollment in a remote HF medication management program for the optimization of GDMT) or (2) the “simultaneous” arm (parallel participation in patient and provider education and enrollment in a remote HF medication management program for the optimization of GDMT). Education consists of curated print and video microlearning content for patients and providers' notification regarding their eligibility for GDMT before active participation in the remote management GDMT clinic. For patients enrolled with LVEF < 50% not on SGLT2i at baseline, we will perform a secondary randomization to test a strategy of medication titration sequencing using SGLT2i first versus traditional titration sequencing. Traditional titration sequencing is based on approval dates of medications and guideline sequencing and follows the order of BB, ARNI/ACEi/ARB, MRA, and SGLT2i. Patients will be stratified by LVEF (≥ 50% and < 50%) and by enrollment category, either outpatient or post‐hospital discharge (defined as within 30 days of discharge) for prespecified secondary analysis. All randomizations will be completed through computer‐generated algorithms using block randomization with block sizes of 30 without stratifying based on LVEF to ensure an equal number of patients in each group. The randomization allocation based on the blocks will be concealed to the study personnel.

### 2.3 Intervention

**Figure 2:**
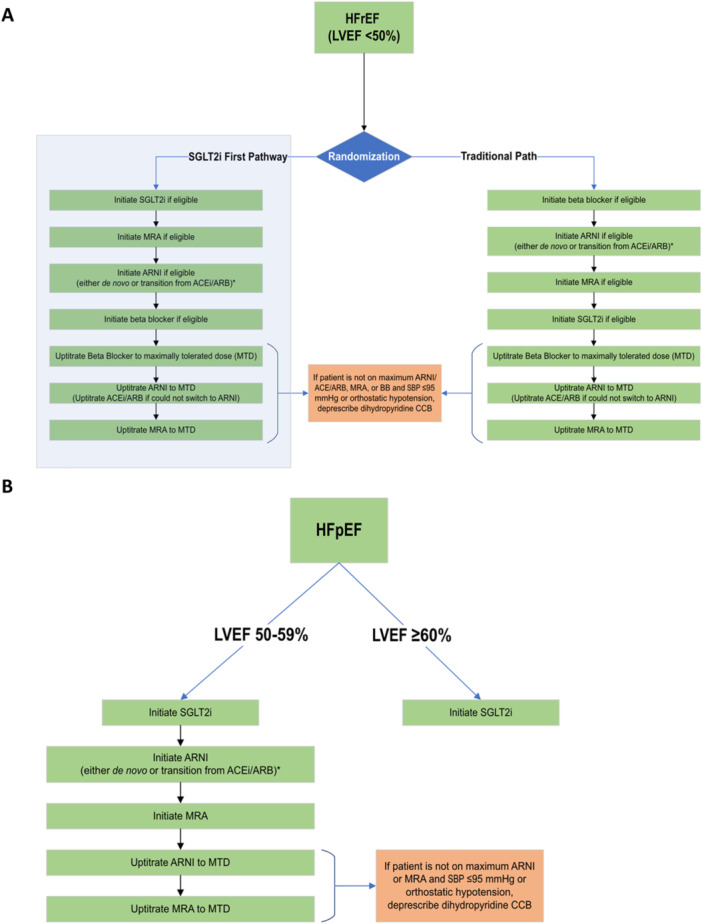
Medication titration algorithm. (A) Medication algorithm for HFrEF, (B) Medication algorithm for HFpEF; SGLT2i (sodium glucose cotransporter‐2 inhibitor), MRA (mineralocorticoid receptor antagonist), ARNI (angiotensin receptor‐neprilysin inhibitor), ACEi (angiotensin‐converting enzyme inhibitor), ARB (angiotensin II receptor blocker), MTD (maximum tolerated dose).

While the patients are enrolled in the remote GDMT optimization program, medication titration is managed under a Collaborative Drug Therapy Management (CDTM) agreement by a pharmacist. A team including pharmacists, advanced practice providers, general cardiologists, and HF specialists developed a medication management algorithm. The algorithm includes initiation, titration, and discontinuation of BB, ARNI, angiotensin converting enzyme inhibitors, angiotensin II receptor blockers, MRA, and SGLT2i based on LVEF categories (Figure 2). Since guidelines for HFpEF incorporating new study data have not been formally published in the United States at the time of study initiation, algorithms were created based on expert consensus documents [17, 18] as well as recent updates to ESC guidelines. Following the same expert consensus documents [17, 18], we set the LVEF threshold for HFpEF as ³50% and added ARNI and MRA to the treatment algorithm for those with an EF 50–59% based on the extended benefits of MRAs and ARNIs in “less‐than‐normal” LVEF [17]. Consequently, the final medication initiation and titration protocol included LVEF categories of < 50%, 50%–59%, and ≥ 60%. These management plans were approved by the BWH Pharmacy and Therapeutics Committee.

We constructed a customer relationship management platform using Microsoft Dynamics 365 [19], which enables automated data integration from the EHR and medication management with follow‐up by a multidisciplinary team. All aspects of the platform comply with the Health Insurance Portability and Accountability Act of 1996 and institutional requirements.

Navigators, a group of nonclinicians specifically trained to approach patients for the purposes of this study, are primarily responsible for communicating with prospective and enrolled participants. Titration of GDMT is performed by a team of navigators and pharmacists supervised by an advanced practice practitioner (APP) and a cardiologist. Based on a predetermined algorithm, successive medication adjustments are determined by a clinical pharmacist and relayed to the navigator. Navigators communicate with program participants via phone for medication adjustments and monitoring of adverse events, blood pressure, and laboratory values. If the patient accepts the proposed medication adjustment, navigators relay this information to the pharmacist, who sends the new prescription to the patient's preferred pharmacy. Cost, side‐effects, hypotension, and/or laboratory changes as barriers to medication class or dose optimization will be collected at the time of medication initiation attempt. If a patient cannot afford a medication, the study team performs prior authorizations, tier exemptions, and referrals to Serving the Health Insurance Needs of Everyone councilors to assist Medicare‐eligible patients in overcoming financial barriers. The study team also aids participants with Patient Assistance Program applications which are income‐based programs help provide access to medication at low or no cost to the patient, or assists patients in signing up for copay cards to increase medication access. If cost limitations persist, this will be documented and communicated to the patient's primary care and/or cardiology team in the EHR. Patients are able to decline suggested GDMT adjustments for any reason, and this will be documented if it occurs. Specific rules governing sequencing and titration of each medication class are provided in Figure 2.

Educational videos, including information regarding HF diagnosis, HF medications, and self‐care for HF, are shared with program participants on a patient engagement and education platform (Mytonomy) without need for registration. For patients with limited access to electronic devices or who are otherwise unable to access the videos, we will send printed educational material that covers the same topics as the educational videos. Providers are sent a message detailing the LVEF group of their patient with an attached medication algorithm that they are recommended to follow.

### 2.4 Outcomes and Follow‐Up

The primary endpoint of the study is the percent of eligible enrolled patients who achieve utilization of recommended pharmacotherapy for HF at 3 months after randomization, which includes (1) prescription utilization of all four drug classes (ARNI/ARB/ACEI, SGLT2i, BB, and MRA) at any dose in patients with LVEF < 50% and (2) prescription of SGLT2i in patients with LVEF ≥ 50%. This binary primary endpoint was chosen for its pragmatic simplicity, applicability across both HFrEF and HFpEF populations, and alignment with current guideline‐directed treatment goals. It offers a clear and clinically meaningful measure of successful implementation of foundational therapy that can be consistently interpreted across HF phenotypes. The prespecified primary, secondary, and exploratory outcomes are summarized in Table 2.

**Table 2:** Study endpoints and outcome measures.

Endpoint category	Specific measure	Definition/details	Time point of assessment (months)
Primary endpoint	Utilization of recommended therapy	Percent of eligible patients achieving: LVEF < 50%: Utilization of four GDMT classes (ARNI/ARB/ACEI, SGLT2i, BB, and MRA). LVEF ≥ 50%: Utilization of an SGLT2i.	3
Secondary endpoint	Any intensification of GDMT	Any initiation or dose titration of a GDMT medication.	6
**Other efficacy and safety endpoints**			
Process metrics	Number of new GDMT medication classes added	Absolute count of new classes initiated.	6
	Percent reaching maximum tolerated dose	Proportion of patients at maximum tolerated dose for each GDMT class.	6
	Percent on ≥ 50% recommended dose	Proportion of patients on at least 50% of the recommended target dose for each class.	6
	Prescription for four optimal‐dose classes	Proportion of patients with LVEF < 50% who have a prescription for all four GDMT classes at optimal dose.	6
	Utilization of four optimal‐dose classes	Proportion of patients with LVEF < 50% with self‐reported utilization of all four GDMT classes at optimal dose.	6
	Prescription for SGLT2i	Proportion of patients with LVEF ≥ 50% with a prescription for an SGLT2i.	6
	Utilization of SGLT2i	Proportion of patients with LVEF ≥ 50% with self‐reported utilization of an SGLT2i.	6
	Prescription for ARNI/MRA/SGLT2i	Proportion of patients with LVEF 50%–59% with a prescription for ARNI, MRA, and SGLT2i.	6
	Utilization of ARNI/MRA/SGLT2i	Proportion of patients with LVEF 50%–59% with self‐reported utilization of ARNI, MRA, and SGLT2i.	6
	Any worsening HF	Composite of worsening NYHA functional class or hospitalization for HF.	6
Surrogate clinical outcomes	Weight change	Change from baseline (kg).	6
	Blood pressure	Change from baseline (mmHg).	6
	NT‐proBNP	Change from baseline (pg/mL).	6
Clinical outcomes (efficacy and safety)	Any worsening HF	Composite of worsening NYHA functional class or hospitalization for HF.	6
	Hypotension	Systolic blood pressure < 90 mmHg with associated symptoms.	6
	Hypo/hyperkalemia	Serum potassium < 3.0 mmol/L or > 6.0 mmol/L.	6
	Acute kidney injury (AKI)	Increase in serum creatinine by ≥ 1.5 times the baseline value.	6
	Any hospitalization	All‐cause hospitalization.	6
	Death	All‐cause mortality.	6
Health utilization outcomes	Emergency department visit	All‐cause and HF‐specific.	6
	Hospital admission	All‐cause and HF‐specific.	6
	*Patient & provider‐reported outcomes*		
	Patient satisfaction	Net promoter score (NPS) survey.	6
	Provider satisfaction	Assessed via survey.	6
Exploratory outcomes	GDMT medication prescriptions	Analysis of prescription patterns at multiple time points.	1, 2, 6, and 12
	Cost‐effectiveness analysis	Economic analysis of the intervention.	12
	Heart failure collaboratory GDMT score	Change from baseline (for patients with LVEF < 50%).	6
	Optimization potential score	Change from baseline (for patients with LVEF < 50%).	6

Abbreviations: ACEI, angiotensin‐converting enzyme inhibitor; AKI, acute kidney injury; ARB, angiotensin II receptor blocker; ARNI, angiotensin receptor‐neprilysin inhibitor; BB, beta‐blocker; GDMT, guideline‐directed medical therapy; HF, heart failure; LVEF, left ventricular ejection fraction; MRA, mineralocorticoid receptor antagonist; NPS, Net Promoter Score; NT‐proBNP, N‐terminal pro‐B‐type natriuretic peptide; NYHA, New York Heart Association; SGLT2i, sodium‐glucose cotransporter‐2 inhibitor.

The secondary endpoint is any intensification of GDMT at 3 months, which includes any initiation or titration of a GDMT medication. Intensification of GDMT, which includes any initiation or titration of a GDMT medication, will be captured as the number of intensification steps and compared between treatment groups. For example, the initiation of any dose of an ACEi, followed by two titrations and the initiation of SGLT2i, would count as four intensification steps. We will then compare the primary and secondary outcomes between groups at 6 months postenrollment after all patients receive the remote HF clinic intervention. Patients will be followed actively for 6 months or to the end of active medication management; however, we will continue to obtain data from EHR for another 6 months and compare outcomes at 12 months postenrollment to assess the durability of the results. An interim analysis will be performed after 100 participants have completed 4 months of the program to assess if any changes to the study design are needed based on efficacy, safety, and operational metrics.

The primary analyses will be performed according to the intention‐to‐treat principle, using data for all randomized patients. The “education‐first” group will be compared with the “simultaneous” medication titration and education groups. Sensitivity analyses will be performed in patients engaged in the program for at least 3 months, and in patients who complete the treatment program.

For the second randomization in participants with HFrEF comparing different sequencing of therapies, we will analyze primary and secondary outcomes by comparing patients in the “SGLT2i‐first” and “traditional pathway” GDMT sequencing groups.

Other efficacy and safety endpoints will include incidence of emergency department visits, hospitalizations, and deaths adjudicated as attributable to adverse drug reactions within our protocols, during study follow‐up in both groups. We will explore the barriers to the initiation or continuation of GDMT, including, but not limited to, cost, adverse events, provider medication changes, and patient compliance and preferences. We will also perform additional analyses for process metrics, surrogate outcomes (weight change, blood pressure, and NT‐proBNP level), traditional clinical outcomes (worsening HF, hospitalization for HF, all‐cause hospitalization, and death), healthcare utilization, and patient satisfaction.

Finally, we will perform prespecified analysis across various subgroups of interest, including age, sex, race, ethnicity, primary insurance, comorbid conditions, type/setting of enrollment (outpatient vs. post‐HF hospitalization), and baseline LVEF.

### 2.5 Statistical Plan

We estimate that the baseline percentage of patients with HF who meet the primary endpoint definition will be ~10% [11]. In the education/notification arm, we anticipate a ~4% absolute improvement to ~14% based on other studies with educational interventions that target patients [11]. Based on our previous experience, we hypothesize that a strategy of a remote, navigator‐led, pharmacist‐driven HF program will increase this proportion to 25% at 3 months after randomization. Our prior pilot study of a similar remote, navigator‐led program formed the basis for this estimate [14]. With 250 patients in each strategy group (500 patients in total) there will be > 85% power to detect a significant difference between groups at a two‐sided 0.05 significance level. We estimate a rate of dropout (patients who stop receiving treatment from the program) of 10%. The primary endpoint (as determined by last observed medication use) will be assessed in all patients.

For the second randomization of the sequence of GDMT therapy initiations in patients with LVEF < 50%, we expect that the percentage of patients in the traditional titration sequencing group who meet the primary endpoint will be 15%. Considering an imbalanced enrollment of patients with HFrEF and HFpEF, with 40% of patients with HFrEF, a total of 100 patients in each group will provide 80% power to detect an 18% difference between groups at a two‐sided 0.05 significance level, with an estimated 10% dropout (patients who stop receiving treatment from the program). This assumption is supported by prior real‐world data demonstrating hyperkalemia rates of approximately 28%–38% with traditional GDMT sequencing, as well as clinical trial data showing that SGLT2 inhibitors reduce the incidence of hyperkalemia and hypotension [20, 21, 22] —two common barriers to the initiation and up‐titration of MRAs and ARNIs [23, 24]. Based on these findings, we believe that initiating therapy with an SGLT2i may mitigate these adverse effects and lead to a higher proportion of patients achieving full GDMT in this arm.

The percentage of subjects who achieve the primary endpoint and other dichotomous secondary outcomes will be compared between the two randomization groups using a two‐sided chi‐squared test. Risk ratios will be calculated as measures of association to express the difference in dichotomous outcomes. Continuous endpoints will be reported as mean and standard deviation or median with the interquartile range depending on the distribution. No adjustment for multiplicity will be made in any of the secondary outcome analyses and, as such, will be interpreted as exploratory.

A planned interim analysis will occur after 100 participants have completed 4 months of the program to evaluate the study progression and operational metrics across the program and will assess if any changes to the study design are needed at that time.

## 3 Discussion

The options for disease‐modifying HF pharmacotherapies have expanded rapidly in the past two decades [1], and multiple lines of evidence now overwhelmingly support their benefit to reduce morbidity and mortality in this high‐risk population, as reflected in contemporary clinical practice guidelines [2, 5, 18]. While the evidence base for these therapies is robust, their translation into clinical practice remains suboptimal. This “implementation gap” is substantial and highlights missed opportunities to avert preventable morbidity, mortality, and financial burden [8, 25, 26, 27]. Multiple barriers to optimal treatment deployment have been identified, including limited provider knowledge and expertise, insufficient provider time to appropriately identify eligible patients and favorably modify treatment, medication costs, and limited access to care [28, 29]. Moreover, the growing complexity of treatment algorithms amplifies these challenges, further hindering rapid integration of these therapies in daily practice.

To date, we have implemented population health programs for the management of hypertension and hyperlipidemia in over 10 000 patients, diabetes in 200 patients, and HFrEF in 197 patients across the MGB healthcare system [14, 30, 31]. Since our initial experience in scaling medication optimization in patients living with HFrEF, significant advances have been made. A newer class of medications, SGLT2‐inhibitors, have demonstrated marked improvements in clinical outcomes not only for patients with diabetes, but also for patients with chronic kidney disease as well as HFrEF and HFpEF [1].

Furthermore, the randomized, international STRONG‐HF trial stressed the overwhelming benefit of rapid up‐titration of GDMT among individuals admitted with acute HF at and after discharge by demonstrating a substantial reduction in the risk of all‐cause death or HF readmission compared to usual care at 180 days [13]. Based on this evidence, the 2023 focused update of the 2021 ESC guidelines for HF included a Class I recommendation for the initiation and rapid up‐titration of GDMT before discharge, followed by frequent and careful follow‐up visits in the first 6 weeks after a HF hospitalization [5]. However, the intensity of resource and time demands in STRONG‐HF make replicating such an approach in a real‐world setting challenging [13]. Indeed, current care delivery systems are not designed to identify and deliver care innovations rapidly and effectively. As such, novel healthcare delivery approaches enabling efficient and widespread achievement of contemporary guideline recommendations are critically needed.

Several interventions have been studied to improve GDMT prescription for patients with HF, including clinician education, EHR‐based BPAs, patient registries, and various HF‐specific care delivery programs. The Patient‐Centered Care Transitions in HF trial [32], which used a translational care model with nurse‐led self‐care education for patients and follow‐up appointments after discharge, did not show any improvement on a composite outcome of all‐cause readmission, ED visit, or death at 3 months [32]. The Care Optimization Through Patient and Hospital Engagement for HF [33] trial used HF and quality improvement experts to educate providers and provide them feedback based on HF process measures but failed to show any significant benefit on reducing time to first HF rehospitalization or death, or in change in a composite HF quality‐of‐care score over 3 years. The Pragmatic Trial Aimed at Improving Use of Guideline Directed Medical Therapy in Outpatients With Heart Failure (PROMPT‐HF) randomized trial was conducted in Yale‐New Haven Health System to study the effect of a BPA delivered through EHR to improve prescriptions for GDMT for HFrEF [9]. This intervention was shown to increase the proportion of patients with HFrEF who had an increase in the number of prescribed GDMT at 30 days (26% in the intervention arm vs. 19% in the usual care arm). Electronically Delivered, Patient‐Activation Tool for Intensification of Medications for Chronic Heart Failure with Reduced Ejection Fraction (EPIC‐HF) which was conducted in the University of Colorado Health system using nudges designed for patients to impact prescriber behavior [11]. In this study, patients with HFrEF were randomized to usual care versus a patient activation intervention that included an educational video and a medication checklist that were sent before cardiology clinic visits. This intervention showed clear benefit in improving intensification of GDMT (i.e., new initiation or dose increase), where it occurred in 49% in the intervention group versus 30% in the usual care group at 30 days.

Finally, the Implementation of Medical Therapy in Hospitalized Patients with Heart Failure with Reduced Ejection Fraction (IMPLEMENT‐HF) study that included hospitalized patients with HFrEF, investigated a virtual care team‐guided strategy to recommend GDMT intensification to primary provider teams compared to usual practice [10]. It showed that this intervention increased the rate of initiation and intensification of GDMT during hospitalization. Although some of these interventions show promise to create impact by making small and easily scalable changes in prescriber and patient‐facing applications, it is not clear whether they can create durable changes in prescription patterns or show more than modest benefit demonstrated in these trials. In addition, these interventions are still anchored to clinical encounters creating a significant limitation for frequency of GDMT initiation/up‐titration that can be achieved in a short amount of time. In considering the entire patient experience, including at home, our intervention does not rely on traditional clinical encounters, creating a significantly higher number of opportunities for GDMT optimization.

Furthermore, instead of creating additional alerts for clinicians, which can lead to alert fatigue, our intervention aims to decrease the burden of clinicians by taking over adverse‐event surveillance after initiation or up‐titration of medications. Finally, studies targeting improvements in HF care predominantly focus on patients with HFrEF, leaving an important opportunity to improve care for the large number of patients for whom new therapies are available; therefore, we designed our intervention to include patients with HF across the LVEF spectrum.

The roll over from education‐first to simultaneous education and medication management at 3 months in a clinical trial of HF is an important aspect of the trial design. The “education‐first” allows for an “enhanced standard of care” with notification of both patients and providers of their eligibility for these medication classes—and education to both patient and provider of the rationale for these recommendations. By allowing patients to roll over to the medication management arm, both providers and patients will not be left without appropriate prescriptions for guideline‐directed medical therapy even if these are not prescribed by their regular care team. It will allow for a period of randomized comparison between groups. HF patients may benefit from an incremental approach to treatment, especially if they are not acutely ill. Starting with education allows patients to gain knowledge about their condition and the importance of adherence to treatment regimens. After this initial period, introducing medication management gradually may help patients better understand and accept the necessity of their medications. This phased approach might enhance patient engagement and long‐term adherence. The roll over at 3 months allows for an assessment of the safety and tolerability of medications, particularly if patients in the education‐first arm are not already on these medications. This analysis can inform decisions about the most effective treatment approach. The analysis at 6 months will assess for the ability of the education‐first group to demonstrate any effects of education pre‐treatment on treatment uptake or adherence to therapy. This period, with enough time to complete full titration with multiple months of buffer, will assess for any differences in uptake or adherence to therapy at what would be a steady state after the initiation and titration period for both groups. Many of the traditional medications used for the treatment of HFrEF can cause hypotension—often limiting further initial titration of GDMT—or hyperkalemia, which also often causes a slowdown or cessation of attempts by providers to continue with GDMT initiation and titrations. By starting therapy with an SGLT2i—a class of medications known to have little effect on blood pressure, as well as a mild reduction in serum potassium, we believe that starting with SGLT2i may mitigate both hyperkalemia and hypotension‐related treatment gaps.

Taken together, improving the implementation of lifesaving therapeutic approaches remains a foundational clinical and research priority in HF. By improving the uptake and utilization of GDMT across the HF spectrum through a lean, digital platform, we aim to improve the quality and quantity of life while reducing costs of hospitalization and disease progression associated with suboptimal care. While the benefits of these therapies are known, methods to improve the implementation of optimal medical care remain unknown. Targeting patient and provider education alone has been insufficient, and focusing on optimizing therapy in inpatients often identifies patients too late and can lead to challenges with medication tolerance and cost. By addressing practical, last‐mile implementation challenges, we believe we can demonstrate methods to guide the uptake and adherence to indicated medical therapies. These learnings will extend not only to implementation in HF and cardiovascular disease but also to other deadly, disabling, and costly chronic medical conditions that substantially impact public health.

## Ethics Statement

The study protocol was approved by the Mass General Brigham Institutional Review Board. All investigations were conducted in accordance with the Declaration of Helsinki.

## Conflicts of Interest

Dr. Alexander J. Blood reports grants from Astra Zeneca, General Electric Health, Boehringer Ingelheim, Eli Lilly, Merck, and Novo Nordisk; personal fees from Alnylam, Astra Zeneca, Boehringer Ingelheim, Novo Nordisk, Milestone Therapeutics, iRhythm, NODE Health, Walgreens Health, Medscape, Color Health, Corcept Therapeutics, Nference Inc., Withings, and Arsenal Capital Partners; and equity in AIwithCare, Knownwell Health, Porter Health, and Signum Technologies. Dr. Ozan Unlu receives funding from the National Heart Lung and Blood Institute (T32HL007604) and reports equity in AIwithCare. Dr. John W. Ostrominski reports research support from the National Institutes of Health (5T32HL007604‐39 and L30HL175757). Dr. Shahzad Hassan received funding from the National Heart Lung and Blood Institute (T32HL007604). Hunter Nichols, PharmD reports serving as a consultant for Recor Medical and as a contractor for Elsevier. Dr. Christopher P. Cannon reports receiving research grants (calendar years 2022‐2024) from Amgen, Better Therapeutics, Boehringer‐Ingelheim (BI), and Novo Nordisk; he receives salary support from Colorado Prevention Center (CPC) Clinical Research, which receives research grant support from Amgen, Bayer, Cleerly, Esperion, Lexicon, Silence; he also reports consulting fees from Amryt/Chiesi, Amgen, Ascendia, Biogen, BI, BMS, CSL Behring, Genomadix, Lilly, Janssen, Lexicon, Milestone, Novartis, Pfizer, and Rhoshan. Dr. Akshay S. Desai reports receiving institutional research grants (to Brigham and Women's Hospital) from Abbott, Alnylam, AstraZeneca, Bayer, DevPro Biopharma, and Novartis; he reports personal consulting fees from Abbott, Alnylam, AstraZeneca, Axon Therapeutics, Bayer, Biofourmis, Boston Scientific, Endotronix, iRhythm Technologies, GlaxoSmithKline, Medpace, Medtronic, Merck, New Amsterdam, Novartis, Parexel, Porter Health, Regeneron, River2Renal, Roche, scPharmaceuticals, Verily, and Zydus. Dr. Benjamin M. Scirica reports institutional research support (to Brigham and Women's Hospital) from Amgen, Better Therapeutics, Boehringer Ingelheim, Foresite Labs, Milestone Pharmaceutical, Merck, NovoNordisk, Pfizer, and Verve Therapeutics; he reports consulting fees from Abbvie (DSMB), Amgen, AstraZeneca (DSMB), Bayer, Boehringer Ingelheim (DSMB), Elsevier Practice Update Cardiology, Hanmi (DSMB), Lexeo (DSMB), NovoNordisk, and Verve Therapeutics; he holds equity in Health at Scale, Arboretum Lifesciences, and AIwithCare; and a family member is an employee at Vertex Pharmaceuticals and holds stock. Samantha Subramaniam, Daniel Gabovitch, Jacqueline Chasse, Marian McPartlin, Christian Figueroa, Emma Collins, Megan Twining, Matthew Varugheese, and Dr. Kavishwar Wagholikar report no relevant disclosures. This study was made possible by Boehringer Ingelheim Pharmaceuticals Inc. (BIPI) and Eli Lilly and Company (Lilly), who provided support for the Study. The authors meet criteria for authorship as recommended by the International Committee of Medical Journal Editors (ICMJE) and were fully responsible for all aspects of the trial and publication development.

## Data Availability

Data sharing is not applicable to this article, as no datasets were generated or analyzed during the current study.
